# Evaluation of the EFNS/PNS diagnostic criteria in a cohort of CIDP patients

**DOI:** 10.1002/acn3.51357

**Published:** 2021-04-07

**Authors:** Diamantis Athanasopoulos, Jeremias Motte, Thomas Grüter, Nuray Köse, Min‐Suk Yoon, Susanne Otto, Christiane Schneider‐Gold, Ralf Gold, Anna L. Fisse, Kalliopi Pitarokoili

**Affiliations:** ^1^ Department of Neurology St. Josef‐Hospital Ruhr‐University Bochum Germany; ^2^ Immunmediated Neuropathies Biobank (INHIBIT) Ruhr‐University Bochum Bochum Germany; ^3^ Department of Neurology Evangelic Hospital Hattingen Hattingen Germany

## Abstract

**Objective:**

To evaluate the European Federation of Neurological Societies (EFNS)/Peripheral Nerve Society (PNS) diagnostic criteria for chronic inflammatory demyelinating polyneuropathy (CIDP) in a cohort of patients diagnosed and treated for CIDP in a tertiary university hospital.

**Methods:**

In a monocentric retrospective study of 203 CIDP patients, diagnosed according to expert opinion, we evaluated the EFNS/PNS diagnostic criteria. Clinical course and nerve conduction studies (NCS) over 1 year from first referral were studied. Secondarily, we compared the clinical and paraclinical characteristics, including nerve ultrasound, of patients who failed with those who fulfilled the criteria in order to identify clinically relevant differences.

**Results:**

At 1 year, 182 (89.7%) patients fulfilled the criteria (156/76.9% definite, 22/10.8% probable, and 4/2% possible). Twenty‐one (10.3%) patients did not because the electrodiagnostic criteria remained negative. These still showed signs of demyelination but did not reach the cut‐off values. They also presented typical, albeit less pronounced, multifocal nerve enlargement in ultrasonography. Mean disability at presentation and 1 year after was significantly lower. Most importantly, a relevant proportion of these patients also responded to therapy (6/21 = 28.6% vs. 82/182 = 45.3% of those fulfilling the criteria).

**Interpretation:**

CIDP diagnosis could be established for 89.7% of patients over the course of 1 year using EFNS/PNS criteria. The remaining patients (10.3%) presented with milder disability, less accentuated demyelination, but otherwise similar characteristics and still considerable probability of treatment response. Failure to fulfill diagnostic criteria should not automatically preclude treatment. Nerve ultrasound should be considered as a complementary diagnostic tool to detect signs of inflammation in CIDP.

## Introduction

Chronic inflammatory demyelinating polyneuropathy (CIDP) is a rare^1^ relapsing/remitting or progressive autoimmune neuropathy with a multifaceted presentation, partially understood pathophysiology, and still unknown etiology. It is, however, one of the best treatable forms of polyneuropathy (PNP), hence an accurate and early diagnosis is highly important in order to achieve favorable patient outcomes.

The diagnosis of CIDP is made primarily on the basis of clinical history and clinical examination combined with the findings of nerve conduction studies (NCS) and supported by other paraclinical methods such as cerebrospinal fluid (CSF) analysis, nerve biopsy, and neuroimaging methods like MRI. Novel diagnostic methods, such as high‐resolution nerve ultrasound (HRUS), have been increasingly used in the last years. There are no pathognomonic signs or findings and no sensitive surrogate markers to clearly differentiate or exclude CIDP.

Since the term CIDP was first coined by Dyck et al. in 1975,^2^ at least 15 different sets of diagnostic criteria with varying diagnostic accuracy have been published.^3^ Initially, the aim of such criteria was to specifically define CIDP cases to facilitate research. Later criteria focused increasingly on clinical application toward recognizing the right patients to treat. In recent years, the European Federation of Neurological Societies (EFNS)/Peripheral Nerve Society (PNS) criteria, first published in 2006^4^ and then revised in 2010,^5^ have been broadly adopted for research purposes.^6^ They have largely replaced the criteria of the Ad Hoc Subcommittee of the American Academy of Neurology (AAN)^7^ due to their increased sensitivity and still high specificity.^8^ These implement clinical, electrodiagnostic, and supportive criteria, including biopsy, CSF analysis, and nerve MRI, to establish the diagnosis of CIDP in three defined levels of confidence: possible, probable, and definite. However, in real‐world conditions outside clinical studies, the adherence to such diagnostic criteria is still often neglected^9^, ^10^ despite good evidence of their usefulness. In the hectic of day to day practice, they are sometimes considered as time consuming. There is evidence from studies which supports that CIDP is often overdiagnosed.^11^, ^12^ Recognized usual pitfalls are an overinterpretation of mildly elevated CSF protein or a liberal interpretation of mild signs of demyelination in NCS. Among experienced clinicians in tertiary centers, however, there is also a common perception that CIDP is being also underdiagnosed, especially in mild cases or by patients with coexisting diabetes.^13^, ^14^ Due to the rarity of the disease and the aforementioned lack of suitable markers, the magnitude of this effect cannot be objectified. Misdiagnosis, however, can in both cases have a detrimental impact on patients, who could be subjected to unwarranted and potentially harmful chronic treatments or conversely deprived of important disease‐modifying therapy.

The purpose of this study is to evaluate the EFNS/PNS CIDP criteria in a cohort of CIDP patients in a real‐world setting. What proportion of patients who received the diagnosis of CIDP on the basis of clinical findings and expertise did fulfill these criteria? We also aim to explore and compare the characteristics of patients who do not fulfill the criteria in order to identify potentially differentiating factors.

## Methods

### Identification of patients

In this monocentric retrospective study, we analyzed the data of patients who were referred to our clinic (St. Josef‐Hospital, University clinic of the Ruhr‐University Bochum) with the suspected diagnosis “immune‐mediated neuropathy” in the years 2010 to 2018. To investigate PNP cases in our clinic, apart from conducting NCS, lumbar puncture, routine blood tests, including vitamin B12 and folic acid, and nerve ultrasound, we routinely perform serologic testing for hepatitis, HIV, syphilis, Lyme borreliosis, antinuclear antibodies, extractable nuclear antigen, dual strand DNA antibodies, antineutrophil cytoplasmic antibodies, rheumatoid factor, paraneoplastic neuronal antigens (including at a minimum antibodies against Hu, Ri, Yo, Ma/Ta, CRMP5, and amphiphysin) as well as serum/urine immunofixation. Antibodies targeting myelin‐associated glycoprotein (MAG) are tested only in cases with IgM gammopathy. Furthermore, at least a chest radiography and ultrasound of the abdomen, as well as prostate‐specific antigen (PSA) for male patients, are performed. Further investigation with computed tomography, urology, gynecology, or rheumatology consultation is performed when deemed necessary by the treating physician. We reviewed the patient files to determine the final diagnosis as it was set by the treating physician. Patients with initial referring diagnosis of immune neuropathy but other final clinical diagnosis, such as diabetic, toxic, and critical illness neuropathy, as well as other clearly defined inflammatory neuropathies like multifocal motor neuropathy (MMN), neuropathy associated with monoclonal IgM and anti‐MAG antibodies (anti‐MAG neuropathy), and paraneoplastic or vasculitic neuropathy were excluded. The cases with final clinical diagnosis of CIDP were identified and included in the study.

### Clinical data collection

Patients’ history as well as laboratory findings were retrospectively evaluated to review the clinical inclusion and exclusion criteria as defined by the EFNS/PNS CIDP criteria in their 2010 revision.^5^

All NCS that were reviewed were performed in our clinic. The electronically archived recordings of NCS from the time of first referral as well as any follow‐up examinations up to a year from that time point were reevaluated. The motor NCS were reviewed according to the major electrodiagnostic EFNS/PNS criteria. The values of distal motor latency (DML), conduction velocity (CV), compound muscle action potential (CMAP) amplitudes as well as duration distally and proximally, and the F‐wave persistence and minimal latency were calculated as proposed by the EFNS/PNS criteria.

The corresponding sensory NCS were also reviewed in light of the supportive criteria. When available, the somatosensory evoked potential (SSEP) studies were also reviewed. With both methods, only measurements with reproducibly elicited sensory nerve action potentials (SNAP) or SSEP were taken into account. If the nerves were not excitable, then this supportive criterion was considered negative.

The available laboratory data were reviewed. Regarding CSF protein, an upper limit of normal of 50 mg/dL (500 mg/L) was set as cut‐off value irrespective of age. A cell count of 10/*µ*L was defined as pleocytosis as per the EFNS/PNS supportive criteria definition. For patients on whom a lumbar puncture was not performed in our clinic, external results of prior CSF examinations were considered using the abovementioned cut‐off values.

For patients on whom a nerve biopsy was performed, the written pathology reports were reevaluated and binary stratified as positive or negative according to the EFNS/PNS criteria recommendations. We considered biopsies showing a predominantly demyelinating chronic neuropathy with sings of demyelination and remyelination as well as endoneurial inflammatory cellular infiltrates as compatible with a CIDP diagnosis.

The reevaluation of available nerve MRI studies was also based on the written radiology reports, which were also characterized as positive or negative. Findings of proximal nerve enlargement and/or enhancement, especially of the nerve roots, cauda equina, or plexuses, were considered as suggestive of CIDP.

In order to evaluate the supportive criterion “response to therapy”, the clinical course over the first year after referral was retrospectively evaluated according to available documentation. The Overall Disability Sum Score (ODSS), which is routinely used in our clinic to assess PNP patients, was chosen as the best possible objective measure of disease severity and disability. Patients who showed at least one point of improvement were categorized as therapy responders, adhering to the strict definition of the EFNS/PNS guidelines, thereafter “EFNS response”. Patients who showed progressive objective (according to ODSS) deterioration without treatment and were stabilized but ODSS did not improve after treatment were categorized as “stabilized” in order to differentiate them from complete lack of a therapeutic effect (“no response”).

Using this data, we determined a diagnosis according to the 2010 revision of the EFNS/PNS CIDP criteria from one of four possible categories: no CIDP, possible CIDP, probable CIDP, and definite CIDP.

Beyond the methods relevant for the EFNS/PNS criteria, we also evaluated the results of HRUS which is routinely performed in our clinic. HRUS of the peripheral nerves is increasingly used as a diagnostic tool in inflammatory neuropathies.^15^, ^16^, ^17^, ^18^, ^19^, ^20^ In particular, multifocal, inhomogeneous predominantly proximal nerve enlargement, measured as an increase of the nerve cross‐sectional area (CSA), is a typical finding in CIDP. Various scores deriving from CSA values have been developed over recent years as a means to diagnose and/or differentiate various inflammatory neuropathies.^15^, ^16^, ^18^, ^19^, ^20^ We evaluated the CSA values of the median, ulnar, and radial nerves in the upper extremities as well as tibial, fibular, and sural nerves in the lower extremities. The brachial plexus was also assessed in the supraclavicular and interscalene spaces. We also calculated the Bochum ultrasound score^20^ (BUS, ranging from 0 to 4 points, with one point given for increased CSA at each of the four following sites: ulnar nerve in Guyon’s canal, ulnar nerve in the upper arm, radial nerve in the spiral groove, and sural nerve between the gastrocnemius muscle), which was developed from our group as a marker of CIDP (if more than two sites show increased CSA, score of 2 or more) in comparison with Guillain–Barré syndrome patients.

### Statistical analysis

Descriptive statistics include counts and percentages for nominal or dichotomous variables, as well as mean and standard deviation for continuous variables. Categorical variables were analyzed using the Fisher’s exact test. Continuous variables were first tested for normality using the Shapiro–Wilk test, then for equality of variances with the Levene’s test. Normally distributed variables were analyzed using the Student’s two‐tailed *t*‐test, and non‐normally distributed variables with the use of the Mann‐Whitney non‐parametric test. Multiple comparisons were performed using the ANOVA and Kruskal–Wallis tests, respectively. *p* < 0.05 was considered significant.

## Results

### Patient characteristics

From 2010 till 2018, a total of 408 patients were referred to our clinic with the presumptive diagnosis “immune‐mediated neuropathy”. Due to lack of neurophysiological data, 17 patients were excluded. After reviewing the final clinical diagnosis and excluding other diagnosis as aforementioned, a total 208 patients with clinically diagnosed CIDP were identified. Of these, five patients who were not followed up were also excluded from the study. The remaining 203 patients all met the clinical inclusion EFNS/PNS criteria for either typical or atypical CIDP. None of these patients met any exclusion criteria. Thirty‐six of 203 patients also had known diabetes at presentation. The clinical characteristics of the cohort can be seen in Table 1.

**Table 1:** Clinical and paraclinical characteristics of the cohort.

	*n* = 203	no.	%
Male		140	69.0
Female		63	31.0
Typical		144	70.9
Atypical		59	29.1
Of these: MADSAM		15	7.4
Mixed motor & sensory		24	11.8
Pure sensory		20	9.9
MGUS		23	11.3
Diabetes		36	17.7
Age at manifestation (mean ± SD in years)		54.8 ± 13.3	
Age at diagnosis (mean ± SD in years)		57.8 ± 13.7	
Years to diagnosis (mean ± SD in years)		3.0 ± 3.7	
ODSS at presentation (mean ± SD)		2.32 ± 1.84	
ODSS after 1 year (mean ± SD)		2.59 ± 1.86	

	(available *n*)		%
Increased CSF protein	(174)	130	74.7
Positive nerve biopsy	(78)	40	51.3
Positive nerve MRI	(4)	2	50.0
Positive SSEP/sNCS criterion	(203)	94	46.3
Treatment response after 1 year^1^	(202^1^)		
EFNS response		88	43.6
Stabilization		81	40.1
No response		33	16.3

MADSAM, multifocal acquired demyelinating sensory and motor neuropathy; MGUS, Monoclonal gammopathy of undetermined significance, excluding patients with IgM gammopathy with anti‐myelin‐associated glycoprotein (MAG) antibodies; ODSS, overall disability sum score; CSF, cerebrospinal fluid; SSEP, somatosensory evoked potential; sNCS, sensory nerve conduction studies; EFNS, European Federation of Neurological Societies.

^1^ One patient died of unrelated causes. For definition of response to therapy, see methods section. SSEP/sNCS criterion as defined by the EFNS/PNS supportive criteria.^5^

### EFNS criteria

The nerves examined by NCS and the frequency at which each nerve fulfilled any of the electrodiagnostic criteria are presented in Table 2.

**Table 2:** Nerves examined with NCS and frequency of fulfillment of the electrodiagnostic demyelination criteria.

Nerve	Of 203 patients	%	% positive								
			Any criterion	I‐A	I‐B	I‐C	I‐D	I‐E	I‐F	I‐G	II
Median	183	90.1	39.5	n.a.	13.0	7.7	4.8	7.7	4.3	20.2	6.3
Ulnar	115	56.7	38.5	11.8	10.3	6.6	4.4	8.1	3.7	19.9	5.9
Tibial	199	98.0	51.7	16.4	7.4	1.5	8.0	13.9	31.8	10.9	n.a.
Peroneal	27	13.3	34.1	11.8	5.9	0.0	11.8	5.9	8.8	8.8	2.9

Criteria as defined by EFNS/PNS^5^: I‐A: Motor distal latency prolongation ≥50% above ULN. I‐B: Reduction of motor conduction velocity ≥30% below LLN. I‐C: Prolongation of F‐wave latency ≥30% above ULN (≥50% if amplitude of distal negative peak CMAP <80% of LLN values). I‐D: Absence of F‐waves if the nerve has a distal negative peak CMAP amplitudes ≥20% of LLN. I‐E: Partial motor conduction block: ≥50% amplitude reduction of the proximal negative peak CMAP relative to distal, if distal negative peak CMAP ≥20% of LLN. I‐F: Abnormal temporal dispersion (>30% duration increase between the proximal and distal negative peak CMAP). I‐G: Distal CMAP duration (interval between onset of the first negative peak and return to baseline of the last negative peak) increase (median ≥6.6 msec, ulnar ≥6.7 msec, peroneal ≥7.6 msec, and tibial ≥8.8 msec). II: ≥30% amplitude reduction of the proximal negative peak CMAP relative to distal, excluding the posterior tibial nerve, if distal negative peak CMAP ≥20% of LLN.

NCS, nerve conduction studies; ULN, upper limit of normal; LLN, lower limit of normal; CMAP, compound muscle action potential.

At presentation, 153 patients (75.4%) met at least one of the EFNS/PNS electrodiagnostic criteria for demyelination in one nerve. Eighty‐three patients (40.9%) met the definite, seven patients (3.5%) the probable, and 63 patients (31.0%) the possible electrodiagnostic criteria. By also applying the supportive criteria at this time point of initial presentation, a diagnosis of CIDP according to the EFNS/PNS criteria (thereafter called EFNS‐CIDP) could be established in these 153 patients: 122 patients (60.1%) were classified as definite, 23 patients (11.3%) as probable and eight patients (3.9%) as possible EFNS‐CIDP. Fifty patients (24.6%) did not meet any electrodiagnostic criteria of demyelination therefore had no EFNS‐CIDP.

Over the course of 1 year, an increasing number of patients met the electrodiagnostic criteria on follow‐up NCS allowing for classification as EFNS‐CIDP. The response to therapy at this time point was evaluated as a supportive criterion. In total, EFNS‐CIDP could be diagnosed in 182 patients (89.7%), thereafter called group A. One hundred and fifty‐six patients (76.9%) had a definite, 22 patients (10.8%) a probable, and four patients (2%) a possible EFNS‐CIDP. Twenty‐one patients (10.3%) still did not meet any electrodiagnostic criteria of demyelination therefore were classified as no EFNS‐CIDP, thereafter called group B.

It is also noteworthy to report that exceeding the prespecified analysis criteria, we reviewed the later course of the 21 patients of group B and found that another seven patients fulfilled the electrodiagnostic EFNS/PNS criteria in the long run (five patients in the second year and another two after 6 years). All of these seven patients would at that point have been classified as definitive EFNS‐CIDP. The remaining 14 patients did not fulfill the criteria at any point during the time they were followed up in our clinic (in average 2.2 years with a minimum of 1 and maximum of 9 years). Of these patients, one fulfilled three supportive criteria, five patients fulfilled two of them, and seven patients only one. One patient did not fulfill any of the supportive criteria. The response rate in the first year of the seven patients who later fulfilled the EFNS/PNS criteria was higher than the remaining 14: EFNS defined response: 4/7 (57.1%) versus 2/14 (14.3%), stabilization: 3/7 (42.9%) versus 8/14 (57.1%), no response: 0/7 (0%) versus 4/14 (28.6%).

### Comparison of groups A and B

#### Clinical characteristics

The mean ODSS at presentation and after 1 year was significantly lower in group B in comparison to group A (*p* < 0.001, *p* = 0.022, respectively). There were no other statistically significant differences between groups A and B in respect to sex/gender, age at first manifestation, age at diagnosis, interval from manifestation to diagnosis, coincidence of diabetes or monoclonal gammopathy, subtype of CIDP, or any of the supportive criteria. More importantly, there was no significant difference regarding the response to therapy. Fewer patients in group B required therapy with cyclophosphamide, rituximab, or bortezomib—this difference was not statistically significant (Table 3).

**Table 3:** Clinical and paraclinical characteristics of groups A and B.

	*n* = 182	Group A	%	*n* = 21	Group B	%	*p*
Male		125	68.7		15	71.4	1.000
Female		57	31.3		6	28.6	–
Typical		128	70.3		16	76.2	0.800
Atypical		54	29.7		5	23.8	0.800
Of these: MADSAM		14	7.7		1	4.8	1.000
Mixed		22	12.1		2	9.5	1.000
Pure sensory		18	9.9		2	9.5	1.000
MGUS		20	11.0		3	14.3	0.714
Diabetes		30	16.5		6	28.6	0.223
Age at manifestation (mean ± SD in years)		54.6 ± 13.2			57 ± 14.4		0.427
Age at diagnosis (mean ± SD in years)		57.5 ± 13.6			60.7 ± 14.1		0.313
Time to diagnosis (mean ± SD in years)		2.9 ± 3.6			3.7 ± 4.3		0.377
ODSS at presentation (mean ± SD)		2.41 ± 1.90			1.52 ± 0.93		**<0.001**
ODSS after 1 year (mean ± SD)		2.66 ± 1.93			2.05 ± 0.97		**0.022**

	(available *n*)		%	(available *n*)		%	*p*
Increased CSF protein	(156)	117	75.0	(18)	13	72.2	0.779
Positive nerve biopsy	(75)	37	49.3	(3)	3	100.0	(0.241)
Positive nerve MRI	(4)	2	50.0	(0)	0		–
Positive SSEP/sNCS criterion	(182)	84	46.2	(21)	10	47.6	1.000
Treatment response after 1 year^1^	(181^1^)			(21)			
EFNS defined response		82	45.3		6	28.6	0.168
Stabilization		70	38.7		11	52.4	0.246
No response		29	16.0		4	19.0	0.755
Treatment^2^							
Steroids		131	72.0		17	81	0.449
IVIg		136	74.7		12	57.1	0.117
Oral Immunosuppressives		74	40.7		8	38.1	1.000
Escalation therapy		29	15.9		2	9.5	0.748

Treatment with steroids was variable. Most usually administered as intravenous pulses, almost exclusive of methylprednisolone, with varying dosages and frequency (250–1000 mg/day for 3 days, every 6 to 12 weeks). Fewer patients were treated with oral prednisolone with an initial dosage of 1 mg/kg body weight followed by tapering.

Escalation therapy was considered any therapy with rituximab, cyclophosphamide, or bortezomib.

For definition of response to treatment, see methods section.

SSEP/sNCS criterion as defined by the EFNS/PNS supportive criteria.^5^

MADSAM, Multifocal acquired demyelinating sensory and motor neuropathy; MGUS, Monoclonal gammopathy of undetermined significance, excluding patients with IgM gammopathy with anti‐myelin‐associated glycoprotein (MAG) antibodies; ODSS, Overall disability sum score; CSF, cerebrospinal fluid; SSEP, somatosensory evoked potential; sNCS, sensory nerve conduction studies; EFNS, European Federation of Neurological Societies; IVIg, intravenous immunoglobulin.

^1^ One patient died of unrelated causes. Significant *p* values marked in bold.

^2^ Maintenance treatment with IVIG was with 1 g/kg of body weight every approximately 4 weeks.

#### Electrophysiological parameters

As expected, group A showed more pronounced signs of demyelination in comparison to group B (Table 4).

**Table 4:** Mean values of nerve conduction study parameters, groups A and B (mean ± SD).

Nerve		*n*	Group A	*n*	Group B	*p*
Motor nerves						
Median	DML	167	4.87 ± 2.08	16	3.93 ± 0.55	0.062
	cMAP‐A	167	4.70 ± 2.37	16	5.21 ± 2.50	0.374
	cMAP‐D	167	6.09 ± 1.70	16	5.56 ± 0.75	0.379
	mCV	167	**44.34** ± **10.96**	16	**50.47** ± **4.89**	**0.029**
	F‐W‐Per	161	67.80 ± 30.97	15	74.33 ± 24.1	0.412
	F‐W‐Lat	149	35.56 ± 21.14	14	29.51 ± 2.71	0.114
Ulnar	DML	107	3.69 ± 1.96	8	3.18 ± 0.39	0.969
	cMAP‐A	107	5.54 ± 2.58	8	6.28 ± 2.04	0.397
	cMAP‐D	107	**6.16** ± **1.92**	8	**4.78** ± **0.70**	**0.003**
	mCV	106	48.45 ± 10.71	8	54.20 ± 6.80	0.142
	F‐W‐Per	91	69.84 ± 35.09	8	83.75 ± 30.20	0.154
	F‐W‐Lat	80	33.49 ± 9.09	8	29.96 ± 3.56	0.163
Tibial	DML	155	**6.48** ± **3.28**	15	**4.39** ± **1.06**	**0.001**
	cMAP‐A	178	2.92 ± 3.00	21	3.75 ± 5.03	0.706
	cMAP‐D	155	6.94 ± 3.12	15	6.24 ± 1.19	0.611
	mCV	148	**37.33** ± **7.00**	15	**41.63** ± **3.53**	**0.008**
	F‐W‐Per	134	53.40 ± 44.77	15	82.83 ± 26.79	0.069
	F‐W‐Lat	89	62.92 ± 10.14	14	57.80 ± 7.39	0.066
Fibular	DML	17	7.66 ± 4.45	0	–	–
	cMAP‐A	26	0.85 ± 1.01	1	0	–
	cMAP‐D	17	7.17 ± 3.77	0	–	–
	mCV	16	34.85 ± 8.91	0	–	–
	F‐W‐Per	13	8.85 ± 14.74	0	–	–
	F‐W‐Lat	5	62.30 ± 3.81	0	–	–
Sensory nerves						
Median	SNAP	137	5.13 ± 7.89	16	4.62 ± 3.35	0.466
	sCV	120	48.15 ± 7.38	14	50.04 ± 10.18	0.387
Ulnar	SNAP	87	**3.15** ± **5.05**	8	**4.50** ± **2.39**	**0.014**
	sCV	70	50.87 ± 8.48	8	54.69 ± 8.92	0.233
Radial	SNAP	5	6.53 ± ± 7.28	0	–	–
	sCV	4	57.65 ± 3.56	0	–	–
Sural	SNAP	123	2.89 ± 3.56	15	3.56 ± 4.56	0.961
	sCV	90	41.93 ± 8.36	8	45.63 ± 7.28	0.175

In cases where both sides were measured, the mean value of those was used.

Significant *p* values marked in bold. *n* = available data in each group.

DML, distal motor latency in msec; cMAP‐A, distal compound motor action potential amplitudes in mV; cMAP‐D, distal compound motor action potential duration in msec; mCV, motor conduction velocity in m/sec; F‐W‐Per, F‐wave persistency in %; F‐W‐Lat, F‐wave latency in msec; SNAP, sensory nerve action potential in *µ*V; sCV, sensory conduction velocity in m/sec.

##### NCS of upper extremity nerves

The mean DML, distal CMAP duration, and F‐wave latencies of the median and ulnar nerves were more prolonged in group A than in group B, and the mean motor and sensory CV were slower. The mean CMAP amplitudes of these nerves were also lower in group A. However, only the SNAP amplitudes of the ulnar nerve were lower, not those of the median nerve. Of these, only differences in the mean distal CMAP duration of the ulnar nerve, the mean CV of the median nerve, and the SNAP amplitudes of the ulnar nerve were statistically significant (*p* = 0.003, *p* = 0.031, and *p* = 0.014, respectively).

##### NCS of lower extremity nerves

The mean DML, distal CMAP duration, and F‐wave latencies of the tibial nerve were more prolonged in group A than in group B, and the mean CV was slower. The CMAP amplitudes were also lower in group A. The differences in mean DML and CV were statistically significant (*p* = 0.001 and *p* = 0.031, respectively).

There were not sufficient measurements of the peroneal nerve in group B, so a comparison was not possible.

The CV of the sural nerve was also slower in group A than in group B, and the SNAP amplitudes were lower. No difference reached statistical significance.

#### HRUS

Both groups presented multifocal and inhomogeneous enlargement in proximal but also distal segments of arm and leg nerves, as well as the brachial plexus. However, the mean CSA of the brachial plexus and of multiple nerves, particularly the proximal median, ulnar as well as radial nerve, was more often enlarged in group A. There was, however, no statistically significant difference in regard to nerve enlargement in HRUS. Furthermore, the BUS was more often positive in group A, without that being statistically significant (Tables 5 and 6).

**Table 5:** Mean nerve CSA in HRUS, groups A and B (mm^2^, mean ± SD).

Nerve	Location	n	Group A	n	Group B	p	Normal values
Median	Carpal tunnel	147	11.10 ± 3.00	15	11.80 ± 3.71	0.567	6.9 ± 2.8
	Forearm	147	8.83 ± 2.99	15	7.42 ± 2.18	0.104	8.0 ± 2.3
	Upper arm	146	11.39 ± 4.04	15	10.40 ± 3.76	0.224	8.4 ± 2.9
Ulnar	Guyon’s canal	146	6.19 ± 1.99	15	5.69 ± 1.80	0.394	5.2 ± 1.0
	Forearm	144	6.43 ± 2.02	15	5.98 ± 1.37	0.526	5.5 ± 1.3
	Elbow	132	9.77 ± 3.81	14	10.02 ± 3.64	0.700	5.3 ± 1.4
	Upper arm	138	8.14 ± 3.09	15	6.89 ± 1.42	0.138	6.5 ± 1.8
Radial	Spiral groove	144	5.92 ± 2.95	15	5.16 ± 1.66	0.393	3.3 ± 1.5
Brachial plexus	Interscalene space	122	42.14 ± 26.37	12	32.38 ± 10.91	0.804	30.9 ± 10.8
	Supraclavicular space	98	67.82 ± 33.32	11	56.16 ± 23.44	0.395	46.1 ± 18.3
Vagus		8	2.44 ± 0.70	2	1.75 ± 0.68	0.400	5.5 ± 1.6
Fibular	Fibular head	139	13.56 ± 4.68	15	12.93 ± 3.52	0.622	7.1 ± 2.3
	Popliteal fossa	122	9.96 ± 4.93	12	8.03 ± 2.96	0.140	8.6 ± 1.7
Tibial	Popliteal fossa	134	20.23 ± 9.28	15	18.66 ± 8.05	0.590	8.4 ± 2.7
	Ankle	138	11.18 ± 4.65	15	10.62 ± 4.02	0.790	6.3 ± 1.5
Sural	Middle of calf	137	2.73 ± 1.48	15	2.30 ± 0.56	0.595	1.8 ± 0.6

*n*, available data in each group.

CSA, cross‐sectional area; HRUS, high‐resolution nerve ultrasound.

Normal values used in our lab as published by Kerasnoudis et al.^26^

**Table 6:** Pathologically increased nerve CSA in HRUS, groups A and B.

Nerve	Location	n	Group A	%	n	Group B	%	p
Median	Carpal tunnel	147	64	43.5	15	6	40.0	1.0000
	Forearm	147	26	17.7	15	1	6.7	0.4695
	Upper arm	146	41	28.1	15	3	20	0.7616
Ulnar	Guyon’s canal	146	45	30.8	15	5	33.3	1.0000
	Forearm	144	38	26.4	15	2	13.3	0.3593
	Elbow	132	93	70.5	14	10	71.4	1.0000
	Upper arm	138	39	28.3	15	1	6.7	0.1178
Radial	Spiral groove	144	58	40.3	15	3	20	0.1661
Brachial plexus	Interscalene space	122	34	27.9	12	2	16.7	0.5133
	Supraclavicular space	98	34	34.7	11	2	18.2	0.3325
Vagus		8	0	0.0	2	0	0.0	1.0000
Fibular	Fibular head	139	103	74.1	15	9	60.0	0.2396
	Popliteal fossa	122	26	21.3	12	1	8.3	0.4584
Tibial	Popliteal fossa	134	107	79.9	15	10	66.7	0.3163
	Ankle	138	107	77.5	15	10	66.7	0.3470
Sural	Middle of calf	137	55	40.1	15	6	40.0	1.0000
BUS ≥2		139	54	38.8	15	4	26.7	0.4138

*n* = available data in each group.

BUS, Bochum ultrasound score, ranging from 0 to 4 points, with one point given for increased CSA at each of the four following sites: ulnar nerve in Guyon’s canal, ulnar nerve in the upper arm, radial nerve in the spiral groove, and sural nerve between the heads of the gastrocnemius muscle.

### Analysis of diagnostic categories

A further analysis of the various subgroups within group A showed that the previously mentioned clinical, electrophysiological, and sonographic differences to group B were more accentuated for patients with definite than those with probable CIDP. The mean ODSS at presentation and at 1 year was significantly lower in group B in comparison to patients with definite CIDP, while this difference was significant only at presentation to patients with probable CIDP. Significant differences in NCS parameters were noted only between group B and patients with definite CIDP. Nerve enlargement in HRUS showed a similar pattern and was more pronounced, but not statistically significant, for patients with definite CIDP. Due to the very small size of the possible CIDP group (*n* = 4), it was excluded from the statistical analysis (Tables S1–S4).

### Analysis of diabetic patients

As mentioned, 36 patients, 17.7% of the cohort, had diabetes. 83.3% of these patients fulfilled the EFNS/PNS criteria for CIDP, only slightly less often than non‐diabetics (91%). These patients were in average older and presented slightly higher disability, a difference which was statistically significant at 1 year. The response to immunotherapy did not significantly differ, although the absolute rate was lower. Diabetics showed less pronounced signs of demyelination in NCS: Mean DML and distal CMAP duration were less prolonged and mean F‐wave persistence was higher, but only the difference in mean CMAP duration of the median and tibial nerves reached statistical significance. Mean CV slowing and mean CMAP amplitudes showed no significant differences. HRUS did not show any significant differences. The mean CSA of proximal arm nerves and especially that of the brachial plexus was larger in non‐diabetics but this did not reach statistical significance due to the wide value distribution (Table 7 and Tables S5 and S6).

**Table 7:** Clinical and paraclinical characteristics of diabetics versus non‐diabetics.

	*n* = 36	Diabetics	%	*n* = 167	Non‐diabetics	%	*p*
HbA1c % (mean ± SD)		6.8 ± 1.33			*n.a*.		
EFNS/PNS CIDP		30	83.3		152	91.0	0.223
Definite		25	69.4		131	78.4	0.277
Probable		5	13.9		17	10.2	0.555
Possible		0	—		4	2.4	—
Male		26	72.2		114	68.3	0.696
Female		10	27.8		53	31.7	0.696
Typical		24	66.7		120	71.9	0.548
Atypical		12	33.3		47	28.1	0.548
Of these: MADSAM		3	8.3		12	7.2	0.733
Mixed		7	19.4		17	10.2	0.151
Pure sensory		2	5.6		18	10.8	0.538
MGUS		4	11.1		19	11.4	1.000
Age at manifestation (mean ± SD in years)		58.6 ± 11.3			52.4 ± 16.2		**0.028**
Age at diagnosis (mean ± SD in years)		62.3 ± 10.9			55.1 ± 16.9		***0.016***
Time to diagnosis (mean ± SD in years)		3.6 ± 3.9			2.7 ± 3.6		0.195
ODSS at presentation (mean ± SD)		2.58 ± 1.90			2.26 ± 1.83		0.339
ODSS after 1 year (mean ± SD)		3.17 ± 2.24			2.47 ± 1.75		**0.041**

	(available *n*)		%	(available *n*)		%	
Increased CSF Protein	(33)	26	78.8	(141)	104	73.8	0.659
Positive nerve biopsy	(20)	12	60	(58)	28	48.3	0.441
Positive nerve MRI	(0)	—	—	(4)	2	50	—
Positive SSEP/sNLG criterion	(36)	16	44.4	(167)	78	46.7	0.855
Treatment response after 1 year^1^	(36)			(166^1^)			
EFNS defined response		13	36.1		75	45.1	0.360
Stabilization		14	38.9		67	40.4	1.000
No response		9	25		24	14.5	0.136
Treatment^2^							
Steroids		25	69.4		123	73.7	0.680
IVIg		24	66.7		124	74.3	0.408
Immunosuppressives		15	41.7		67	40.1	0.854
Escalation therapy		6	16.7		25	15	0.800

SSEP/sNCS criterion as defined by the EFNS/PNS supportive criteria.^5^

MADSAM, multifocal acquired demyelinating sensory and motor neuropathy; MGUS, Monoclonal gammopathy of undetermined significance, excluding patients with IgM gammopathy with anti‐myelin‐associated glycoprotein (MAG) antibodies; ODSS, overall disability sum score; CSF, cerebrospinal fluid; SSEP, somatosensory evoked potential; sNCS, sensory nerve conduction studies; EFNS, European Federation of Neurological Societies; IVIg, intravenous immunoglobulin.

^1^ One patient died of unrelated causes. Significant *p* values marked in bold.

^2^ Escalation therapy was considered any therapy with rituximab, cyclophosphamide, or bortezomib. For further details on treatment, see Table 3. For definition of response to treatment, see Methods section.

## Discussion

We show that the majority (89.7%) of the patients who were diagnosed with CIDP in a tertiary setting did meet the EFNS/PNS criteria for CIDP. This contrasts with previous reports which examined the criteria in the general neurology praxis and attribute this finding to the complexity of the criteria and the strict electrophysiological criteria for demyelination.^10^, ^11^ However, we have to point out that even in our tertiary center some patients were initially treated as CIDP, although the criteria were only fulfilled later during the following 1 or 2 years. It is only fair to assume that the increased specialization and level of expertise present in a tertiary center, which enable often follow‐up examinations, are partly responsible for this difference. Also, selective filtration of suspect cases through other tiers of the medical system could be leading proportionally more CIDP patients to reach tertiary centers, which is naturally expected since complicated and serious cases would prompt such a referral.

Our result sets, on the one hand, the concern of overdiagnosis and overtreatment partly to rest, at least as far as specialized centers are concerned. On the other hand, it confirms the high sensitivity of the EFNS/PNS criteria but only with frequent follow‐up examinations. However, considering the ever‐increasing demand and high cost of the resource‐intensive treatment with immunoglobulins, a reliable CIDP diagnosis is of high importance.

EFNS/PNS criteria are broadly used for research purposes, as in clinical trials strict adherence to such criteria is essential in order to facilitate a homogenous and robust study population. For everyday clinical application though, the fulfillment or not of such criteria at the first examination should not be the sole deciding factor for the initiation of treatment.

In our study, a considerable portion of 10.3% of the patients (group B) did not meet the EFNS/PNS criteria for the diagnosis of CIDP and the main reasons can be described as follows:

Firstly, the major electrophysiological signs of demyelination were less pronounced in group B and did not reach the cut‐off values of the criteria. This proportion (10.3%) of criteria negative patients is in par with previously published studies. Rajabally et al. in their study to initially validate the EFNS/PNS criteria reported a sensitivity of 81.3% for definite and probable CIDP^8^; in our study, 87.7% of the patients could be diagnosed with definite or probable EFNS/PNS CIDP.
Secondly, group B presented with lower overall disability as measured with the ODSS score and this remained so after 1 year. Overall, it seems these patients represent a group with a milder and/or slower advancing, less aggressive disease. The overall response to therapy did not significantly differ and although not statistically significant, their treatment required less often a therapy with rituximab, cyclophosphamide, or bortezomib. However, we have to point out the limitations of these statistical comparisons due to the small size of group B.

One third of these patients did actually meet the EFNS/PNS criteria in future routine examinations, possibly only then reaching the strict threshold of the electrodiagnostic demyelination criteria. Therefore, early diagnosis of CIDP for patients with no extensive signs of demyelination seems to be a pitfall of EFNS/PNS criteria.

Overall, group B did not differ significantly in any other way from group A. Strictly abiding to the EFNS/PNS criteria would have made the CIDP diagnosis impossible and would have excluded these patients from treatment. Therefore, as previously mentioned, failing to fulfill the EFNS/PNS criteria should not automatically preclude treatment and further consultation in a specialty center should be sought.

On a next level, we proceeded to investigate whether easily applicable novel imaging methods, such as the HRUS, could have detected signs of inflammation (as a CSA increase) for these patients in an early stage of the disease and with minor signs of electrophysiological demyelination.

Even though the electrodiagnostic EFNS/PNS criteria were not met, group B showed indeed typical morphological changes in HRUS, though not as pronounced as in group A. The implementation of HRUS, as an adjunct to NCS, could aid in diagnosing CIDP in such cases.^19^, ^21^ The development of a sensitive and easy to implement diagnostic HRUS algorithm is the object of the ongoing investigation from several groups^15^, ^19^ including present authors, especially in recognition of the fact that an extensive HRUS nerve examination is a time intensive method. The integration of HRUS in future diagnostic criteria seems promising and requires further investigation.

An obvious limitation of our study is the retrospective nature of it, and hence the intrusion of selection bias cannot be excluded. Also limiting is the fact that the NCS were not standardized and different nerves were examined for different patients. All NCS were implemented according to each physician’s discretion and differential diagnostic considerations. However, it is also reassuring that despite this limitation the majority of patients fulfilled the criteria. The tibial nerve was almost always examined, the fibular very rarely. On the upper extremities, the median nerve was examined more often than the ulnar. In group A, the right median and ulnar nerves were examined significantly more often than in group B (Table 8). We cannot rule out the possibility that this difference in the quantity of examined nerves could be the reason why some patients in group B failed to fulfill the electrodiagnostic criteria. We note, however, that from the seven patients who fulfilled them later on, the measurement of a new nerve helped to do that in only one case. In the remaining six patients, signs of worsened demyelination were found in already previously examined nerves. Rajabally et al.^8^ demonstrated that by following a more extensive electroneurographic examination protocol, the sensitivity increases (up to 96.7%) in the expense, however, of decreasing the specificity. Hence, thorough NCS at initial presentation are important for an early diagnosis.

**Table 8:** Nerves examined with nerve conduction studies per group.

Nerve	Group A		Group B		*p*
	*n*	%	*n*	%	
Right median	**68**	**37.4**	**2**	**9.5**	**0.014**
Left median	147	80.8	16	76.2	0.572
Right ulnar	**49**	**26.9**	**1**	**4.8**	**0.030**
Left ulnar	102	56.0	11	52.4	0.819
Right tibial	169	92.9	20	95.2	1.000
Left tibial	140	76.9	15	71.4	0.591
Right fibular	12	6.6	0	0.0	0.618
Left fibular	24	13.2	1	4.8	0.481
Any median	**170**	**93.4**	**16**	**76.2**	**0.020**
Any ulnar	117	64.3	11	52.4	0.341
Any tibial	177	97.3	21	100.0	1.000
Any fibular	28	15.4	1	4.8	0.322
Any median and any tibial	165	90.7	16	76.2	0.059
Median on both sides	45	24.7	2	9.5	0.171
Ulnar on both sides	34	18.7	1	4.8	0.135
Tibial on both sides	132	72.5	14	66.7	0.311
Fibular on both sides	8	4.4	0	0.0	1.000

*n* = Number of patients in each group by which the respective nerve or nerve combination was examined using nerve conduction studies. Significantly different values are given in bold.

Another possible confounding factor could be the inclusion of diabetic patients. There is great controversy and debate on the association of diabetes with CIDP,^13^, ^14^, ^22^, ^23^ especially since diabetic neuropathy can present with demyelinating characteristics and there is no clear‐cut method of distinguishing this from CIDP. Recent studies have shown evidence to support an increased prevalence of CIDP in diabetics.^13^, ^24^ As our study represents a real‐world situation, we chose to include these patients with previously known diabetes who the clinicians considered having comorbid CIDP. We find that the prevalence of diabetes in the cohort (17.7%) is not exceptionally high, considering the mean age and male predominance, and it is compatible with the estimated prevalence of diabetes in the German population of this age group.^25^ Furthermore, the mean HbA1c of our diabetic patients was relatively low (6.8%), indicating a relatively good glycemic control, a finding which should prompt a clinician to further investigate the presence of another cause of neuropathy even in the clinical context of established diabetic neuropathy when new progressive/relapsing symptoms arise. Our patients with diabetes were in average older than non‐diabetics and had relatively higher disability, characteristics comparable with findings of previous studies.^22^, ^24^ NCS showed slightly less accentuated signs of demyelination in diabetics, but only the difference in mean CMAP duration was statistically significant. The most important finding was that fulfillment of the EFNS/PNS criteria as well as treatment response did not differ significantly, so that a substantial proportion of the diabetics benefited from immunotherapy. This goes to show that diabetes should not automatically preclude further investigation or treatment, and that clinicians should maintain a high level of suspicion in order to compensate for this bias.

## Conclusions

EFNS/PNS criteria confirmed CIDP in the majority of suspected cases over the course of 1 year. The criteria failed to diagnose CIDP in a subgroup of patients, despite them responding equally often to therapy and showing similar HRUS abnormalities. This indicates that the decision to treat should not solely be based on fulfillment of the diagnostic criteria. Patients not reaching the cut‐off electrophysiological demyelinating values, such as patients with early disease, can still fulfill the criteria in later examinations. Novel imaging methods, such as HRUS, could assist in an early CIDP diagnosis.

## Conflict of Interest

Diamantis Athanasopoulos, Susanne Otto, and Nuray Köse have no conflict of interest to report. Jeremias Motte received travel grants from Biogen idec, Novartis AG, Teva, and Eisai GmbH, and his research is funded by Klaus Tschira Foundation and Ruhr‐University, Bochum (FoRUM‐program), none related to this work. Thomas Grüter received travel reimbursement from Sanofi Genzyme and Biogen Idec, none related to this work. Min‐Suk Yoon has received speaker honoraria from CSL Behring and Grifols, a scientific grant from CSL Behring, none related to this manuscript. Christiane Schneider‐Gold has received consulting and speaker's honoraria from Alexion Pharmaceuticals, Amicus Therapeutics, Bayer Schering, CSL Behring, Grünenthal, Lupin Pharmaceuticals, and TEVA, none related to this manuscript. Ralf Gold has received consultation fees and speaker honoraria from Bayer Schering, Biogen idec, Merck Serono, Novartis, Sanofi‐Aventis, and TEVA. He also acknowledges grant support from Bayer Schering, Biogen idec, Merck Serono, Sanofi‐Aventis, and TEVA, none related to this work. Anna Lena Fisse received research funding by Georgius Agricola Stiftung Ruhr, received honoraria and travel grants from Novartis AG, Sanofi, and Eisai GmbH, none related to this work. Owns shares of Fresenius SE & Co., Gilead Sciences, Medtronic PLC, and Novartis AG. Kalliopi Pitarokoili received travel funding and speaker honoraria from Biogen Idec, Novartis, and Bayer Schering Pharma and funding from the Ruhr‐University, Bochum (FORUM‐Program), none related to this work.

## Author Contributions

All authors have read and approved the manuscript. Diamantis Athanasopoulos: acquisition, analysis and interpretation of data, and drafting/revising the manuscript for content. Jeremias Motte: acquisition, analysis and interpretation of data, and drafting/revising the manuscript for content. Thomas Grüter: acquisition, analysis and interpretation of data, and drafting/revising the manuscript for content. Nuray Köse: acquisition, analysis and interpretation of data, and revising the manuscript for content. Min‐Suk Yoon: Critical comments during data collection, drafting, and manuscript revision. Susanne Otto: Critical comments during data collection, drafting, and manuscript revision. Christiane Schneider‐Gold: Critical comments during data collection, drafting, and manuscript revision. Ralf Gold: Critical comments during data collection, drafting, and manuscript revision. Anna Lena Fisse: First idea, acquisition, analysis and interpretation of data, drafting and manuscript revision, and study supervision. Kalliopi Pitarokoili: First idea, acquisition, analysis and interpretation of data, drafting and manuscript revision, and study supervision.

## Data Sharing Statement

The data that support the findings of this study are available from the corresponding author upon reasonable request.

## Ethical Standards

The retrospective study was approved from the ethics committee of Ruhr University Bochum vote‐no. 18‐6407.

## Supporting information


**Table S1**. Clinical and paraclinical characteristics of subgroups.
**Table S2**. Mean values of nerve conduction study parameters of subgroups (mean ± SD).
**Table S3**. Mean nerve CSA in HRUS of subgroups (mm^2^, mean ± SD).
**Table S4**. Pathologically increased nerve CSA in HRUS of subgroups.
**Table S5.** Mean values of nerve conduction study parameters of diabetics versus non‐diabetics (mean ± SD).
**Table S6.** Mean nerve CSA in HRUS, diabetics versus non‐diabetics (mean ± SD, *n* = available data in each group).
