# Supplementary material for: Evaluation of the EFNS/PNS diagnostic criteria in a cohort of CIDP patients
Source: Ann Clin Transl Neurol. 2021 Apr 7;8(5):1110–21. doi: 10.1002/acn3.51357 (PMC8108415; doi:10.1002/acn3.51357)
Supplement: Supplementary file 1 — Table S1. Clinical and paraclinical characteristics of subgroups. Table S2. Mean values of nerve conduction study parameters of subgroups (mean ± SD). Table S3. Mean nerve CSA in HRUS of subgroups (mm2, mean ± SD). Table S4. Pathologically increased nerve CSA in HRUS of subgroups. Table S5. Mean values of nerve conduction study parameters of diabetics versus non‐diabetics (mean ± SD). Table S6. Mean nerve CSA in HRUS, diabetics versus non‐diabetics (mean ± SD, n = available data in each group). [file ACN3-8-1110-s001.docx]

| Supplementary Table 1. Clinical and paraclinical characteristics of subgroups | | | | | | | | | | | | |  |
| --- | --- | --- | --- | --- | --- | --- | --- | --- | --- | --- | --- | --- | --- |
|  | ***Definite*** | | | ***Probable*** | | | ***Possible*** | | | ***No CIDP*** | | | ***p*** |
|  | *n=156* | | *%* | *n=22* | | *%* | *n=4* | | *%* | *n=21* | | *%* |  |
| male |  | 110 | 70.5 |  | 12 | 54.5 |  | 3 | 75 |  | 15 | 71.4 | 0.473 |
| female |  | 46 | 29.5 |  | 10 | 45.5 |  | 1 | 25 |  | 6 | 28.6 | 0.473 |
| typical |  | 109 | 69.9 |  | 15 | 68.2 |  | 4 | 100 |  | 16 | 76.2 | 0.808 |
| atypical |  | 47 | 30.1 |  | 7 | 31.8 |  | 0 | 0 |  | 5 | 23.8 | 1.000 |
| of these:  MADSAM |  | 12 | 7.7 |  | 2 | 9.1 |  | 0 | 0 |  | 1 | 4.8 | 0.922 |
| mixed |  | 18 | 11.5 |  | 4 | 18.2 |  | 0 | 0 |  | 2 | 9.5 | 0.699 |
| pure sensory |  | 17 | 10.9 |  | 1 | 4.5 |  | 0 | 0 |  | 2 | 9.5 | 0.433 |
| MGUS |  | 16 | 10.3 |  | 4 | 18.2 |  | 0 | 0 |  | 3 | 14.3 | 0.5661 |
| Diabetes |  | 25 | 16.0 |  | 5 | 22.7 |  | 0 | 0 |  | 6 | 28.6 | 0.396 |
| Age at manifestation  (mean±SD) |  | 54.6 ±13.1 |  |  | 56.3 ±14.5 |  |  | 45.5 ±4.4 |  |  | 57 ±14.4 |  | 0.655 |
| Age at  diagnosis  (mean±SD) |  | 57.6 ±13.6 |  |  | 57.7 ±15.3 |  |  | 52.5 ±7.9 |  |  | 60.7 ±14.1 |  | 0.629 |
| Years to diagnosis  (mean±SD) |  | 3 ±3.5 |  |  | 1.3 ±1.7 |  |  | 7 ±8.4 |  |  | 3.7 ±4.3 |  | 0.081 |
| ODSS at  presentation  (mean±SD) |  | **  2.42 ±1.94 |  |  | *  2.73 ±1.91 |  |  | 1.5 ±1 |  |  | ** / *  1.52 ±0.93 |  | ** <0.01  * <0.05 |
| ODSS after 1 year  (mean±SD) |  | *  2.64 ±1.90 |  |  | 2.81 ±2.09 |  |  | 2.5 ±2.52 |  |  | *  2.05 ± 0.97 |  | * <0.05 |
|  | available n | | | available n | | | available n | | | available n | | |  |
| Increased CSF Protein | 137 | 108 | 78.8 | 16 | 9 | 56.3 | 3 | 0 | 0 | 18 | 13 | 72.2 | 0.125 |
| Positive nerve biopsy | 65 | 34 | 52.3 | 8 | 3 | 37.5 | 2 | 0 | 0 | 3 | 3 | 100 | 0.143 |
| Positive nerve MRI | 4 | 2 | 50.0 | 0 | 0 | - | 0 | 0 | - | 0 | 0 |  | - |
| Positive SSEP/sNCS criterion | 156 | ***  81 | 51.9 | 22 | *** / *  3 | 13.6 | 4 | 0 | 0 | 21 | *  10 | 47.6 | *** <0.001  * <0.05 |
| Treatment Response after 1 year ^†^ | 156 |  |  | 21 |  |  | 4 |  |  | 21 |  |  |  |
| EFNS defined response |  | 74 | 47.4 |  | 8 | 38.1 |  | 0 | 0 |  | 6 | 28.6 | 0.111 |
| stabilization |  | 57 | 36.5 |  | 10 | 47.6 |  | 3 | 75 |  | 11 | 52.4 | 0.199 |
| no response |  | 25 | 16.0 |  | 3 | 14.3 |  | 1 | 1 |  | 4 | 19.0 | 0.872 |
| Treatment: |  |  |  |  |  |  |  |  | 75 |  |  |  |  |
| Steroids |  | 115 | 76.7 |  | 13 | 65 |  | 3 | 75 |  | 17 | 81 | 0.390 |
| IVIg |  | 116 | 77.3 |  | 14 | 70 |  | 3 | 75 |  | 12 | 57.1 | 0.289 |
| Immunosuppresives |  | 62 | 41.3 |  | 10 | 50 |  | 2 | 50 |  | 8 | 38.1 | 0.912 |
| Escalation therapy* |  | 25 | 16.7 |  | 3 | 15 |  | 1 | 25 |  | 2 | 9.5 | 0.753 |

| **Supplementary Table 2. Mean values of nerve conduction studies parameters of subgroups (mean ± SD)** | | | | | | | | | | |
| --- | --- | --- | --- | --- | --- | --- | --- | --- | --- | --- |
| ***Nerve*** |  | ***n*** | ***Definite*** | ***n*** | ***Probable*** | ***n*** | ***Possible*** | ***n*** | ***No CIDP*** | ***p*** |
| **Median** | DML | 142 | 4.94 ±2.18 | 20 | 4.63 ±1.57 | 4 | 4.05 ±0.65 | 16 | 3.93 ±0.55 | 0.163 |
|  | cMAP-A | 142 | 4.69 ±2.38 | 20 | 4.95 ±2.48 | 4 | 4.95 ±1.37 | 16 | 5.21 ±2.50 | 0.621 |
|  | cMAP-D | 142 | 6.15 ±1.73 | 20 | 5.71 ±1.48 | 4 | 5.23 ±0.91 | 16 | 5.56 ±0.75 | 0.218 |
|  | mCV | 142 | 44.09 ±11.15 | 20 | 45.57 ±10.27 | 4 | *50.18* ±5.72 | 16 | 50.47 ±4.89 | 0.072 |
|  | F-W-Per | 137 | 67.80 ±31.09 | 19 | 68.03 ±28.90 | 4 | 83.75 ±21.36 | 15 | 74.33 ±24.1 | 0.680 |
|  | F-W-Lat | 126 | 36.29 ±22.62 | 19 | 31.99 ±9.77 | 4 | 29.4 ±3.75 | 14 | 29.51 ±2.71 | 0.055 |
| **Ulnar** | DML | 93 | 3.77 ±2.07 | 12 | 3.23 ±0.89 | 2 | 2.8 ±0.28 | 8 | 3.18 ±0.39 | 0.765 |
|  | cMAP-A | 93 | 5.52 ±2.65 | 12 | 5.69 ±2.31 | 2 | 5.5 ±0.71 | 8 | 6.28 ±2.04 | 0.700 |
|  | cMAP-D | 93 | **  6.25 ±2.03 | 12 | 5.55 ±0.80 | 2 | 5..95 ±1.34 | 8 | **  4.78 ±0.70 | **  0.006 |
|  | mCV | 92 | 48.17 ±11.14 | 12 | 51.22 ±7.52 | 2 | 44.8 ±0.14 | 8 | 54.20 ±6.80 | 0.288 |
|  | F-W-Per | 77 | 67.11 ±36.26 | 12 | 82.29 ±24.62 | 2 | 100 ±0 | 8 | 83.75 ±30.2 | 0.188 |
|  | F-W-Lat | 66 | 34.10 ±9.2 | 12 | 30.04 ±8.95 | 2 | 31.85 ±0.78 | 8 | 29.96 ±3.56 | 0.105 |
| **Tibial** | DML | 133 | **  6.65 ±3.44 | 17 | 5.37 ±1.92 | 4 | 5.51 ±1.54 | 15 | **  4.39 ±1.06 | **  0.002 |
|  | cMAP-A | 146 | 2.83 ±3.45 | 21 | 4.25 ±6.82 | 4 | 4.29 ±4.9 | 21 | 3.75 ±5.03 | 0.909 |
|  | cMAP-D | 133 | *  7.13 ±3.25 | 17 | *  5.32 ±1.51 | 4 | 6.54 ±0.95 | 15 | 6.24 ±1.19 | *  0.011 |
|  | mCV | 126 | *  37.33 ±7.04 | 17 | 38.16 ±7.21 | 4 | 33.84 ±5.92 | 15 | *  41.63 ±3.53 | *  0.026 |
|  | F-W-Per | 115 | 52.47 ±44.51 | 14 | 61.43 ±47.69 | 4 | 65.63 ±47.19 | 15 | 82.83 ±26.79 | 0.143 |
|  | F-W-Lat | 77 | 63.36 ±10.34 | 9 | 58.04 ±7.12 | 3 | 66.18 ±11.06 | 14 | 57.80 ±7.39 | 0.091 |
| **Fibular** | DML | 15 | 8.02 ±4.63 | 2 | 4.95 ±0.64 | 0 | - | 0 | - | 0.294 |
|  | cMAP-A | 17 | 1.10 ±1.04 | 4 | 0.85 ±1.06 | 0 | - | 1 | 0 | 0.864 |
|  | cMAP-D | 15 | 7.42 ±3.92 | 2 | 5.25 ±1.91 | 0 | - | 0 | - | 0.368 |
|  | mCV | 14 | 34.54 ±9.05 | 2 | 37 ±10.75 | 0 | - | 0 | - | 0.729 |
|  | F-W-Per | 11 | 9.55 ±15.88 | 2 | 5 ±7.07 | 0 | - | 0 | - | 1.000 |
|  | F-W-Lat | 4 | 63.25 ±3.66 | 1 | 58.50 ±0 | 0 | - | 0 | - | 0.329 |
| **Median** | SNAP | 116 | 5.07 ±7.82 | 17 | 5.29 ±9.05 | 4 | 6.2 ±6.29 | 16 | 4.62 ±3.35 | 0.624 |
|  | sCV | 103 | 47.82 ±7.33 | 14 | 48.66 ±7.59 | 3 | 56.93 ±1.74 | 14 | 50.04 ±10.18 | 0.581 |
| **Ulnar** | SNAP | 73 | *  3.09 ±4.86 | 12 | 3.68 ±6.59 | 2 | 2.35 ±3.32 | 8 | *  4.50 ±2.39 | *  0.048 |
|  | sCV | 59 | 50.85 ±8.59 | 10 | 50.87 ±8.70 | 1 | 52 ±0 | 8 | 54.69 ±8.92 | 0.496 |
| **Radial** | SNAP | 4 | 7.3 ±8.17 | 1 | 3.45 ±0 | 0 | - | 0 | - | 0.800 |
|  | sCV | 3 | 59.2 ±2.14 | 1 | 53 ±0 | 0 | - | 0 | - | 0.500 |
| **Sural** | SNAP | 103 | 3.13 ±3.79 | 16 | 1.70 ±1.54 | 4 | 1.45 ±1.80 | 15 | 3.56 ±4.56 | 0.584 |
|  | sCV | 77 | 41.94 ±8.53 | 11 | 42.02 ±7.6 | 2 | 41.08 ±10.57 | 8 | 45.63 ±7.28 | 0.599 |

| **Supplementary Table 3. Mean nerve CSA in HRUS of subgroups (mm2, mean ± SD).** | | | | | | | | | | | |
| --- | --- | --- | --- | --- | --- | --- | --- | --- | --- | --- | --- |
| **Nerve** | **Location** | **n** | **Definite** | **n** | **Probable** | **n** | **Possible** | **n** | **No CIDP** | **p** | **normal values** |
| **Median** | Carpal tunnel | 128 | 11.13 ±3.04 | 15 | 11.48 ±2.70 | 4 | 8.65 ±1.95 | 15 | 11.80 ±3.71 | 0.593 | 6.9 ±2.8 |
|  | Forearm | 128 | 8.90 ±3.08 | 15 | 8.06 ±2.05 | 4 | 9.53 ±3.22 | 15 | 7.42 ±2.18 | 0.075 | 8.0 ±2.3 |
|  | Upper arm | 127 | 11.53 ±4.15 | 15 | 10.27 ±3.35 | 4 | 11.04 ±2.85 | 15 | 10.40 ±3.76 | 0.200 | 8.4 ±2.9 |
| **Ulnar** | Guyon’s canal | 127 | 6.22 ±2.01 | 15 | 5.99 ±2.10 | 4 | 5.81 ±1.14 | 15 | 5.69 ±1.80 | 0.567 | 5.2 ±1.0 |
|  | Forearm | 125 | 6.53 ±2.08 | 15 | 5.96 ±1.55 | 4 | 5.21 ±0.73 | 15 | 5.98 ±1.37 | 0.429 | 5.5 ±1.3 |
|  | Elbow | 116 | 9.83 ±3.86 | 12 | 10.10 ±3.89 | 4 | 7.12 ±0.54 | 14 | 10.02 ±3.64 | 0.947 | 5.3 ±1.4 |
|  | Upper arm | 119 | 8.36 ±3.07 | 15 | 7.40 ±2.70 | 4 | 4.33 ±2.33 | 15 | 6.89 ±1.42 | 0.091 | 6.5 ±1.8 |
| **Radial** | Spiral groove | 125 | 6.03 ±3.08 | 15 | 4.97 ±1.64 | 4 | 6.21 ±2.02 | 15 | 5.16 ±1.66 | 0.399 | 3.3 ±1.5 |
| **Brachial plexus** | Intrascalene space | 106 | 42.47 ±27.32 | 13 | 39.79 ±21.43 | 3 | 40.78 ±9.23 | 12 | 32.38 ±10.91 | 0.801 | 30.9 ±10.8 |
|  | Supraclavicular space | 84 | 68.61 ±33.74 | 11 | 60.08 ±32.05 | 3 | 73.90 ±32.43 | 11 | 56.16 ±23.44 | 0.526 | 46.1 ±18.3 |
| **Vagus** |  | 5 | 2.33 ±0.61 | 1 | 2.98 ±0 | 2 | 2.46 ±1.27 | 2 | 1.75 ±0.68 | 0.407 | 5.5 ±1.6 |
| **Fibular** | Fibular head | 120 | 13.28 ±3.98 | 15 | 15.61 ±8.64 | 4 | 14.07 ±2.26 | 15 | 12.93 ±3.52 | 0.742 | 7.1 ±2.3 |
|  | Popliteal fossa | 104 | 10.10 ±5.07 | 14 | 8.75 ±4.17 | 4 | 10.72 ±3.95 | 12 | 8.03 ±2.96 | 0.201 | 8.6 ±1.7 |
| **Tibial** | Popliteal fossa | 115 | 20.33 ±9.09 | 15 | 20.22 ±11.47 | 4 | 17.19 ±7.19 | 15 | 18.66 ±8.05 | 0.748 | 8.4 ±2.7 |
|  | Ankle | 119 | 11.09 ±4.49 | 15 | 11.25 ±5.87 | 4 | 13.62 ±4.85 | 15 | 10.62 ±4.02 | 0.901 | 6.3 ±1.5 |
| **Sural** | Middle of calf | 119 | 2.73 ±1.48 | 14 | 2.80 ±1.65 | 4 | 2.44 ±0.77 | 15 | 2.30 ±0.56 | 0.839 | 1.8 ±0.6 |

| **Supplementary table 4. Pathologically increased nerve CSA in HRUS of subgroups.** | | | | | | | | | | | | | | |
| --- | --- | --- | --- | --- | --- | --- | --- | --- | --- | --- | --- | --- | --- | --- |
|  |  | **Definite** | | | **Probable** | | | **Possible** | | | **No CIDP** | | |  |
| **Nerve** | **Location** | **n** |  | **%** | **n** |  | **%** | **n** |  | **%** | **n** |  | **%** | **p** |
| **Median** | Carpal tunnel | 128 | 54 | 42.2 | 15 | 10 | 66.7 | 4 | 0 | 0.0 | 15 | 6 | 40.0 | 0.100 |
|  | Forearm | 128 | 23 | 18.0 | 15 | 1 | 6.7 | 4 | 2 | 50.0 | 15 | 1 | 6.7 | 0.147 |
|  | Upper arm | 127 | 36 | 28.3 | 15 | 4 | 26.7 | 4 | 1 | 25.0 | 15 | 3 | 20 | 0.976 |
| **Ulnar** | Guyon’s canal | 127 | 37 | 29.1 | 15 | 6 | 40.0 | 4 | 2 | 50.0 | 15 | 5 | 33.3 | 0.613 |
|  | Forearm | 125 | 35 | 28.0 | 15 | 3 | 20.0 | 4 | 0 | 0.0 | 15 | 2 | 13.3 | 0.506 |
|  | Elbow | 116 | 84 | 72.4 | 12 | 8 | 66.7 | 4 | 1 | 25.0 | 14 | 10 | 71.4 | 0.246 |
|  | Upper arm | 119 | 36 | 30.3 | 15 | 3 | 20.0 | 4 | 0 | 0.0 | 15 | 1 | 6.7 | 0.147 |
| **Radial** | Spiral groove | 125 | 52 | 41.6 | 15 | 4 | 26.7 | 4 | 2 | 50.0 | 15 | 3 | 20 | 0.275 |
| **Brachial plexus** | Intrascalene space | 106 | 30 | 28.3 | 13 | 4 | 30.8 | 3 | 0 | 0.0 | 12 | 2 | 16.7 | 0.737 |
|  | Supraclavicular space | 84 | 30 | 35.7 | 11 | 3 | 27.3 | 3 | 1 | 33.3 | 11 | 2 | 18.2 | 0.717 |
| **Vagus** |  | 5 | 0 | 0.0 | 1 | 0 | 0.0 | 2 | 0 | 0.0 | 2 | 0 | 0.0 | - |
| **Fibular** | Fibular head | 120 | 87 | 72.5 | 15 | 12 | 80.0 | 4 | 4 | 100.0 | 15 | 9 | 60.0 | 0.468 |
|  | Popliteal fossa | 104 | 22 | 21.2 | 14 | 3 | 21.4 | 4 | 1 | 25.0 | 12 | 1 | 8.3 | 0.724 |
| **Tibial** | Popliteal fossa | 115 | 93 | 80.9 | 15 | 11 | 73.3 | 4 | 3 | 75.0 | 15 | 10 | 66.7 | 0.476 |
|  | Ankle | 119 | 94 | 79.0 | 15 | 9 | 60.0 | 4 | 4 | 100.0 | 15 | 10 | 66.7 | 0.200 |
| **Sural** | Middle of calf | 119 | 48 | 40.3 | 14 | 6 | 42.9 | 4 | 1 | 25.0 | 15 | 6 | 40.0 | 1.000 |
| **BUS** ≥**2** |  | 121 | 48 | 39.7 | 14 | 4 | 28.6 | 4 | 2 | 50 | 15 | 4 | 26.7 | 0.659 |

| **Supplementary Table 05.**  **Mean values of nerve conduction studies parameters of diabetics vs non diabetics (mean ± SD).** | | | | | | |
| --- | --- | --- | --- | --- | --- | --- |
| ***Nerve*** |  | ***n*** | ***diabetics*** | ***n*** | ***non diabetics*** | ***p*** |
| **Median** | DML | 34 | 4.3±1.19 | 148 | 4.9±2.16 | 0.159 |
|  | cMAP-A | 35 | 4.7±2.0 | 148 | 4.8±2.47 | 0.860 |
|  | cMAP-D | 35 | **5.5±1.22** | 148 | **6.2±1.71** | **0.051** |
|  | mCV | 35 | 45.5±8.76 | 148 | 44.7±11.14 | 0.976 |
|  | F-W-Per | 32 | 78±21.24 | 144 | 66.2±32.28 | 0.114 |
|  | F-W-Lat | 32 | 33.4±9.52 | 131 | 35.4±22.15 | 0.741 |
| **Ulnar** | DML | 20 | 3.5±0.87 | 95 | 3.7±2.05 | 0.315 |
|  | cMAP-A | 20 | 5.5±3.21 | 95 | 5.6±2.4 | 0.704 |
|  | cMAP-D | 20 | 5.9±1.06 | 95 | 6.1±2.03 | 0.973 |
|  | mCV | 20 | 49±11.46 | 94 | 48.8±10.43 | 0.911 |
|  | F-W-Per | 18 | 79.2±32.78 | 81 | 69.1±35.16 | 0.150 |
|  | F-W-Lat | 17 | 36.2±11.74 | 71 | 32.5±7.85 | 0.251 |
| **Tibial** | DML | 25 | 5.5±1.58 | 145 | 6.4±3.39 | 0.401 |
|  | cMAP-A | 36 | 3.3±4.19 | 163 | 2.9±4.11 | 0.605 |
|  | cMAP-D | 25 | **5.7±1.54** | 145 | **7.1±3.14** | **0.004** |
|  | mCV | 25 | 38.7±8.51 | 138 | 37.5±6.54 | 0.993 |
|  | F-W-Per | 22 | 72.6±41.74 | 127 | 53.6±44.08 | 0.030 |
|  | F-W-Lat | 18 | 61.6±9.23 | 85 | 62.4±10.13 | 0.920 |
| **Fibular** | DML | 2 | 4.5±0.78 | 15 | 8.1±4.57 | 0.590 |
|  | cMAP-A | 5 | 0.4±0.66 | 22 | 0.9±1.06 | 0.284 |
|  | cMAP-D | 2 | 6.1±1.27 | 15 | 7.3±3.99 | 1.000 |
|  | mCV | 2 | 35.7±1.7 | 14 | 34.7±9.55 | 0.890 |
|  | F-W-Per | 2 | 0 | 11 | 10.5±15.57 | 0.410 |
|  | F-W-Lat | 0 | - | 5 | 62.3±3.81 | - |
| **Median** | SNAP | 28 | 4.8±4.66 | 125 | 5.1±8.06 | 0.762 |
|  | sCV | 24 | 47.6±8.54 | 110 | 48.5±7.53 | 0.608 |
| **Ulnar** | SNAP | 17 | 3.9±6.95 | 78 | 3.1±4.36 | 0.965 |
|  | sCV | 14 | 51.6±7.95 | 64 | 51.2±8.73 | 0.883 |
| **Radial** | SNAP | 1 | 4.8 | 4 | 7±8.33 | 1.000 |
|  | sCV | 1 | 61.6 | 3 | 56.3±2.93 | 0.500 |
| **Sural** | SNAP | 19 | 2.8±4 | 119 | 3±3.63 | 0.630 |
|  | sCV | 13 | 42.8±6.91 | 85 | 42.1±8.53 | 0.786 |

| **Supplementary Table 06.**  **Mean nerve CSA in HRUS, diabetics vs non-diabetics (mean ± SD, n= available data in each group).** | | | | | | | |
| --- | --- | --- | --- | --- | --- | --- | --- |
| ***Nerve*** | ***Location*** | ***n*** | ***diabetics*** | ***n*** | ***Non-diabetics*** | ***p*** | ***Ref. range*** |
| **Median** | Carpal tunnel | 28 | 11,23 ±2,90 | 135 | 11,10 ±3,13 | 0.836 | 6,9 ±2,8 |
|  | Forearm | 28 | 8,03 ±2,22 | 135 | 8,76 ±3,07 | 0.232 | 8,0 ±2,3 |
|  | Upper arm | 28 | 10,48 ±2,84 | 134 | 11,40 ±4,21 | 0.269 | 8,4 ±2,9 |
| **Ulnar** | Guyon’s canal | 28 | 5, 97 ±2,36 | 134 | 6,15 ±1,89 | 0.656 | 5,2 ±1,0 |
|  | Forearm | 28 | 6,01 ±1,58 | 132 | 6,45 ±2,03 | 0.281 | 5,5 ±1,3 |
|  | Elbow | 24 | 10,26 ±3,46 | 123 | 9,69 ±3,82 | 0.495 | 5,3 ±1,4 |
|  | Upper arm | 26 | 7,38 ±2,23 | 128 | 8,19 ±3,13 | 0.210 | 6,5 ±1,8 |
| **Radial** | Spiral groove | 28 | 5,23 ±2,11 | 133 | 5,94 ±2,98 | 0.233 | 3,3 ±1,5 |
| **Brachial plexus** | Intrascalene space | 24 | 35,21 ±17,02 | 112 | 42,17 ±26,82 | 0.112 | 30,9 ±10,8 |
|  | Supraclavicular space | 19 | 59,0 ±27,83 | 92 | 67,27 ±33,49 | 0.379 | 46,1 ±18,3 |
| **Vagus** |  | 0 | - | 12 | 2,33 ±0,66 | n.a. | 5,5 ±1,6 |
| **Fibular** | Fibular head | 28 | 13,15 ±3,85 | 127 | 13,46 ±4,79 | 0.754 | 7,1 ±2,3 |
|  | Popliteal fossa | 22 | 8,79 ±2,73 | 114 | 9,95 ±5,07 | 0.299 | 8,6 ±1,7 |
| **Tibial** | Popliteal fossa | 28 | 20,32 ±8,47 | 123 | 19,89 ±9,31 | 0.825 | 8,4 ±2,7 |
|  | Ankle | 27 | 10,39 ±3,35 | 127 | 11,32 ±4,76 | 0.337 | 6,3 ±1,5 |
| **Sural** | Middle of calf | 27 | 2,59 ±1,42 | 126 | 2,68 ±1,42 | 0.778 | 1,8 ±0,6 |
